# Antibiotic Prescribing for Uncomplicated Acute Bronchitis Is Highest in Younger Adults

**DOI:** 10.3390/antibiotics6040022

**Published:** 2017-10-27

**Authors:** Larissa Grigoryan, Roger Zoorob, Jesal Shah, Haijun Wang, Monisha Arya, Barbara W. Trautner

**Affiliations:** 1 Department of Family and Community Medicine, Baylor College of Medicine, Houston, TX 77098, USA; roger.zoorob@bcm.edu (R.Z.); haijunw@bcm.edu (H.W.); 2 Baylor College of Medicine, Houston, TX 77030, USA; jrshah@bcm.edu; 3 Section of Infectious Diseases, Department of Medicine, Baylor College of Medicine, Houston, TX 77030, USA; monishaa@bcm.edu (M.A.); trautner@bcm.edu (B.W.T.); 4 Houston VA Center for Innovations in Quality, Effectiveness and Safety (IQuESt), Michael E. DeBakey Veterans Affairs Medical Center, Houston, TX 77030, USA

**Keywords:** antimicrobial stewardship, antimicrobial resistance, bronchitis, primary care

## Abstract

Reducing inappropriate antibiotic prescribing is currently a global health priority. Current guidelines recommend against antibiotic treatment for acute uncomplicated bronchitis. We studied antibiotic prescribing patterns for uncomplicated acute bronchitis and identified predictors of inappropriate antibiotic prescribing. We used the Epic Clarity database (electronic medical record system) to identify all adult patients with acute bronchitis in family medicine clinics from 2011 to 2016. We excluded factors that could justify antibiotic use, such as suspected pneumonia, COPD or immunocompromising conditions. Of the 3616 visits for uncomplicated acute bronchitis, 2244 (62.1%) resulted in antibiotic treatment. The rates of antibiotic prescribing were similar across the years, *p* value for trend = 0.07. Antibiotics were most frequently prescribed in the age group of 18–39 years (66.9%), followed by the age group of 65 years and above (59.0%), and the age group of 40–64 years (58.7%), *p* value < 0.001. Macrolides were significantly more likely to be prescribed for younger adults, while fluoroquinolones were more likely to be prescribed for patients 65 years or older. Duration of antibiotic use was significantly longer in older adults. Sex and race were not associated with antibiotic prescribing. Our findings highlight the urgent need to reduce inappropriate antibiotic use for uncomplicated acute bronchitis, particularly in younger adults.

## 1. Introduction

Reducing inappropriate antibiotic prescribing is currently a global health priority. The 2015 United States National Action Plan for Combatting Antibiotic Resistant Bacteria set a goal to reduce inappropriate antibiotic use by 50% in outpatient settings, where the majority of antibiotic prescriptions occur [1]. Of the 47 million inappropriately prescribed antibiotics annually in outpatient settings, approximately 34 million stem from acute respiratory infections, therefore, targeting these infections is at the center of this reform movement [2,3,4]. Adhering to treatment guidelines for acute bronchitis could eliminate 7.8 million unnecessary prescriptions annually [2]. As over half of unnecessary prescribing for bronchitis occurs in adult patients, the adult population is a key target of antibiotic stewardship efforts for acute bronchitis [2,3].

As per guidelines from the American College of Physicians and American Academy of Family Physicians, antibiotic treatment is not indicated for uncomplicated acute bronchitis [5,6]. Antibiotic treatment for acute bronchitis may be appropriate in patients with chronic lung disease (e.g., chronic obstructive pulmonary disease (COPD), or emphysema), or patients who have a higher risk for pneumonia (e.g., immunocompromised) [7]. Previous studies indicated that 65–71% of acute bronchitis visits have inappropriately resulted in antibiotic prescriptions [8,9]. These previous studies used survey data to estimate the burden of inappropriate antibiotic use between the years of 1996–2010 [8] and 2010–2011 [9]. Our study adds to the previous literature by studying more recent antibiotic prescribing patterns for uncomplicated acute bronchitis using electronic medical records data from 2011 to 2016, and by excluding factors that could justify antibiotic use such as suspected pneumonia (e.g., chest X-ray orders or abnormal vital signs) or immunocompromising conditions. We also explored the duration and type of antibiotics inappropriately prescribed in primary care settings as well as the relationship between patient characteristics and antibiotic prescribing for acute bronchitis.

## 2. Results

Our study included 3616 visits representing 3134 unique patients over the period of 2011–2016. Most of the visits for uncomplicated acute bronchitis were made by the age group 40–64 years (Table 1).

Of the 3616 visits, in 2244 (62.1%) an antibiotic was prescribed. Of the 1469 excluded visits with a chest X-ray, 961 (65.4%) resulted in antibiotic treatment. Broad-spectrum antibiotics accounted for 97.8% of all antibiotic prescriptions. Macrolides (mostly azithromycin) were prescribed in 87.0% of all prescriptions, followed by amoxicillin-clavulanate (5%), fluoroquinolones (3.3%), and amoxicillin (2.3%). The remaining 2.3% of antibiotics included trimethoprim-sulfamethoxazole, cephalosporins, and clindamycin. The mean duration in days of prescribed azithromycin was 5.1 (standard deviation (SD) 0.6). The mean duration in days of amoxicillin-clavulanate prescription was 9.8 (SD 1.0).

The rates of antibiotic prescribing for bronchitis were similar across the years, *p* value for trend = 0.07 (Table 1). Antibiotics were most frequently prescribed in the age group of 18–39 years (66.9%), followed by age group 65 years and above (59.0%), and age group 40–64 years (58.7%), *p* value < 0.001 (Table 2). The mean duration in days of prescribed antibiotics was 5.7 (SD 1.7) in the age groups of 18–39 years, 5.9 (1.8) in the age group of 40–64 years and 6.0 (1.9) in the age group of ≥65 years (*p* value = 0.02).

Macrolides were more likely to be prescribed for younger adults, while fluoroquinolones were more likely to be prescribed for older patients (≥65 years). Duration of antibiotic prescription was significantly longer in older adults Sex was not associated with antibiotic prescribing in the logistic regression analysis (OR 1.0, 95% CI 0.9–1.2). Race was also not associated with antibiotic prescribing in the logistic regression analysis (*p* value = 0.6).

## 3. Discussion

In our study, antibiotics were commonly prescribed for uncomplicated acute bronchitis in patients visiting family medicine clinics, despite evidence-based guidelines recommending against this practice [5,6]. The antibiotic prescribing rate for bronchitis was similar over the six-year study period (2011–2016). Most visits for uncomplicated acute bronchitis were made by middle-aged adults (40–64 years), but antibiotic prescribing was the highest in younger adults (18–39 years). These findings highlight the urgent need to reduce inappropriate antibiotic use for bronchitis in primary care, particularly in younger adults. Additionally, older adults, who are at particular risk of adverse effects of fluoroquinolones, such as tendon rupture [10] or QT prolongation [11], were more likely to receive fluoroquinolones, highlighting a need to educate primary care providers on the potential for serious side effects from fluoroquinolone use.

Earlier studies showed that antibiotic prescribing for acute bronchitis is common in primary care [8,9]. In a recent United States (U.S.) study using national surveillance data, bronchitis and viral pneumonia had the highest inappropriate antibiotic prescribing compared with other respiratory infections among adult patients presenting in emergency departments [12]. Between 2001 and 2010, utilization of antibiotics for acute respiratory infections remained stable in adult patients aged 20–64 years [12], a finding consistent with our lack of change in prescribing rates over the 5 years of our study. To our knowledge, our study is the first study in the U.S. to report that younger adults (18–39 years) had disproportionately high receipt of antibiotics for uncomplicated acute bronchitis. Studies performed in Spain [13] and Ireland [14] also documented higher rates of outpatient antibiotic prescribing for respiratory tract infections in younger adults.

A limitation of our study is that it was conducted in private family medicine clinics with predominantly white population with private insurance. Therefore, our findings may not be representative of the overall U.S. population. Another limitation is that we performed a database study and not a manual chart review, thus we could not review patient symptoms documented in free text that may have influenced antibiotic prescribing decisions.

Our study adds to the previous literature by examining more recent data (2011–2016) and by excluding factors that could justify antibiotic use such as suspected pneumonia (e.g., chest X-ray orders or abnormal vital signs), presence of immunocompromising conditions, and additional diagnosis for which antibiotics might be indicated. Furthermore, our study adds information on antibiotic choice, and duration of antibiotics prescribed, and the relationship between patient characteristics and antibiotic prescribing. We found that young, healthy adults are more often treated with antibiotics for acute bronchitis, despite the national guidelines recommending against this practice. Therefore, this group represents an important target for antimicrobial stewardship.

Our findings call for implementation of strategies to reduce inappropriate antibiotic use in adult patients with acute bronchitis. Implementation of a decision support strategy for acute bronchitis reduced the overuse of antibiotics in a recent cluster-randomized trial in primary care [15]. Other proposed strategies for reducing antibiotic prescribing in outpatient settings included provider and patient education, delayed prescribing, and prescribing restrictions [16]. Inappropriate antibiotic prescribing for acute respiratory infections was reduced after clinicians displayed a poster in the examination room that included a letter from the clinician to their patients committing to prescribing antibiotics appropriately [17]. In conclusion, despite national attention to the problem of antibiotic overuse, unnecessary antibiotic prescribing remains common in uncomplicated acute bronchitis in primary care. These findings highlight the urgent need to reduce inappropriate antibiotic use for uncomplicated acute bronchitis, particularly in younger adults.

## 4. Materials and Methods

### 4.1. Ethics

The study was conducted in accordance with the Declaration of Helsinki, and the protocol was approved by the Ethics Committee of Baylor College of Medicine on 20 June 2017 (H-39705).

### 4.2. Setting and Study Population

**Figure 1:**
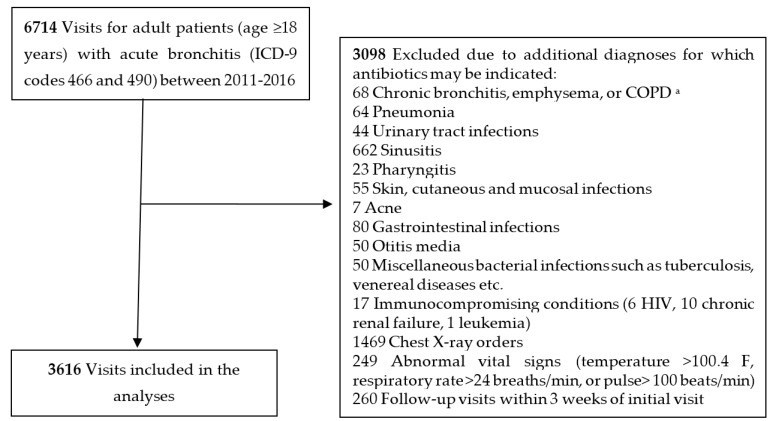
Selection process used to determine visits with uncomplicated acute bronchitis. ^a^ COPD: chronic obstructive pulmonary disease.

The study included two private family medicine clinics in a large urban area. Approximately 19,777 patients attend 3248 appointments at these clinics each month. Patients in both clinics are predominantly female (58%) and white (54%). De-identified records were extracted from the Epic Clarity database (electronic health record system). We selected all patients 18 years and older during the period of January 2011–August 2016 who had any of the two *International Classification of Diseases, Ninth Revision* (ICD-9) codes for acute bronchitis listed as a diagnosis: 466.0 (acute bronchitis and bronchiolitis) and 490.0 (bronchitis, not specified as acute or chronic). Figure 1 shows the selection process used to determine visits with uncomplicated acute bronchitis.

Patient visits were excluded from analysis if they had any of the following additional diagnosis codes for which antibiotics might be indicated: chronic bronchitis, emphysema or COPD, pneumonia, urinary tract infections, sinusitis, pharyngitis, skin, cutaneous and mucosal infections, acne, gastrointestinal infections, otitis media and miscellaneous bacterial infections, as well as visits with chest X-ray orders or abnormal vital signs (temperature >100.4 F, respiratory rate >24 breaths/min, or pulse >100 beats/min) that could be consistent with pneumonia. Presence of immunocompromising conditions (e.g., HIV, active malignancies, and chronic renal failure) was an additional exclusion criterion. Lastly, follow-up visits for bronchitis within 3 weeks of initial visit were excluded in order to prevent duplicating visits of a single episode.

For each eligible visit, we extracted the following variables: patient age, sex, race, date of visit, type of antibiotic prescribed, and duration of treatment. The type of antibiotic was classified as either “narrow-spectrum” or “broad-spectrum”. Amoxicillin, cephalexin, trimethoprim-sulfamethoxazole, lincomycin derivatives (clindamycin) were categorized as “narrow-spectrum”. Amoxicillin-clavulanate, macrolides (azithromycin, clarithromycin, and erythromycin), 2nd to 4th generation cephalosporins, and fluoroquinolones were categorized as “broad-spectrum”.

### 4.3. Statistical Analysis

Descriptive statistics were performed on the general characteristics of acute bronchitis visits, the type of antibiotics prescribed, and duration of treatment in each visit. We studied the trends in the prescribing of antibiotics over the years (2011–2016) using the Mantel–Haenszel χ^2^ test for trend. We used the χ^2^ test to compare antibiotic prescribing between different patient age groups. Logistic regression analyses were used to determine independent predictors of antibiotic prescribing for acute bronchitis. Factors were regarded as being significant in the regression analyses when they had a *p* value of <0.05. Analyses were carried out using SPSS (version 24; SPSS, Chicago, IL, USA).

## Figures and Tables

**Table 1:** Characteristics of all visits for acute uncomplicated bronchitis with and without antibiotic prescriptions, 2011–2016.

	Visit Characteristics Number of Visits (%), *n* = 3616	Number of Visits with Antibiotics Prescribed (% of All Visits; (95% CIs)), *n* = 2244	*p* Value ^d^
Age			**<0.001**
18–39 years, n (%)	1451 (40.1)	971 (66.9; (64.5–69.3))	
40–64 years, n (%)	1662 (46.0)	976 (58.7; (56.3–61.1))	
≥65 years, n (%)	503 (13.9)	297 (59.0; (54.6–63.4))	
Female	2313 (64.0)	1443 (62.4; (60.4–64.4))	0.59
Race ^a^			0.64
White	2288 (74.1)	1408 (61.5; (59.5–63.5))	
Black	515 (16.7)	328 (63.7; (59.4–67.9))	
Other ^b^	286 (9.2)	508 (62.5; (59.1–65.8))	
Year of visit ^c^			0.07 ^e^
2011	673 (18.6)	484 (71.9; (68.4–75.3))	
2012	749 (20.7)	506 (67.6; (64.1–70.9))	
2013	687 (19.0)	348 (50.7; (46.8–54.5))	
2014	559 (15.5)	320 (57.2; (53.0–61.4))	
2015	564 (15.6)	311 (55.1; (50.9–59.3))	
2016 ^c^	384 (10.6)	275 (71.6; (66.8–76.1))	

Data are presented as no. (%) or mean ± standard deviation. CI confidence intervals. ^a^ *n* = 3089, data missing for 527 visits; ^b^ Includes Asian, native Hawaiian, American Indian or Alaska Native; ^c^ Each year represents a full 12 months, except for 2016, which represents January–August; ^d^ *p* value refers to χ^2^ tests comparing antibiotic prescribing between different groups; ^e^ *p* value refers to Mantel–Haenszel χ^2^ test for trend. Boldface indicates a significant result.

**Table 2:** Comparison of antibiotic classes between different age groups for adults with acute uncomplicated bronchitis treated with antibiotics, 2011–2016.

	Visit Characteristics				
	Total (All Ages) n = 3616	18–39 Years n = 1451	40–64 Years n = 1662	≥65 Years n = 503	*p* Value ^a^
No. visits with antibiotics prescribed (%)	2244 (62.1)	971 (66.9)	976 (58.7)	297 (59.0)	
Macrolides	1953 (87.0)	864 (89.0)	841 (86.2)	248 (83.5)	**0.03**
Fluoroquinolones	74 (3.3)	14 (1.4)	44 (4.5)	16 (5.4)	**<0.001**
Other antibiotics	217 (9.7)	93 (9.6)	91 (9.3)	33 (11.1)	0.65

^a^ *p* values refer to χ^2^ tests comparing treatment characteristics between different age groups. Boldface indicates a significant result.

## References

[1] The White House National Action Plan for Combating Antibiotic-Resistant Bacteria. https://obamawhitehouse.archives.gov/sites/default/files/docs/national_action_plan_for_combating_antibotic-resistant_bacteria.pdf.

[2] The Pew Charitable Trusts Antibiotic Use in Outpatient Settings. http://www.pewtrusts.org/~/media/assets/2016/05/antibioticuseinoutpatientsettings.pdf.

[3] The National Committee for Quality Assurance (NCAA) The Healthcare Effectiveness Data and Information Set (HEDIS). http://www.ncqa.org/report-cards/health-plans/state-of-health-care-quality/2016-table-of-contents/acute-bronchitis.

[4] Suda K.J., Hicks L.A., Roberts R.M., Hunkler R.J., Danziger L.H. (2013). A national evaluation of antibiotic expenditures by healthcare setting in the United States, 2009. J. Antimicrob. Chemother..

[5] Harris A.M., Hicks L.A., Qaseem A. (2016). Appropriate antibiotic use for acute respiratory tract infection in adults. Ann. Intern. Med..

[6] Albert R.H. (2010). Diagnosis and treatment of acute bronchitis. Am. Fam. Physician.

[7] Smith S.M., Fahey T., Smucny J., Becker L.A. (2014). Antibiotics for acute bronchitis. Cochrane Database Syst. Rev..

[8] Barnett M.L., Linder J.A. (2014). Antibiotic prescribing for adults with acute bronchitis in the United States, 1996–2010. JAMA.

[9] Fleming-Dutra K.E., Hersh A.L., Shapiro D.J., Bartoces M., Enns E.A., File T.M., Finkelstein J.A., Gerber J.S., Hyun D.Y., Linder J.A. (2016). Prevalence of inappropriate antibiotic prescriptions among US ambulatory care visits, 2010–2011. JAMA.

[10] Arabyat R.M., Raisch D.W., McKoy J.M., Bennett C.L. (2015). Fluoroquinolone-associated tendon-rupture: A summary of reports in the Food and Drug Administration's adverse event reporting system. Expert Opin. Drug Saf..

[11] Mason J.W. (2017). Antimicrobials and QT prolongation. J. Antimicrob. Chemother..

[12] Donnelly J.P., Baddley J.W., Wang H.E. (2014). Antibiotic utilization for acute respiratory tract infections in U.S. emergency departments. Antimicrob. Agents Chemother..

[13] Malo S., Bjerrum L., Feja C., Lallana M.J., Moliner J., Rabanaque M.J. (2015). Compliance with recommendations on outpatient antibiotic prescribing for respiratory tract infections: The case of Spain. Basic Clin. Pharmacol. Toxicol..

[14] Murphy M., Bradley C.P., Byrne S. (2012). Antibiotic prescribing in primary care, adherence to guidelines and unnecessary prescribing—An Irish perspective. BMC Fam. Pract..

[15] Gonzales R., Anderer T., McCulloch C.E., Maselli J.H., Bloom F.J., Graf T.R., Stahl M., Yefko M., Molecavage J., Metlay J.P. (2013). A cluster randomized trial of decision support strategies for reducing antibiotic use in acute bronchitis. JAMA Intern. Med..

[16] Drekonja D.M., Filice G.A., Greer N., Olson A., MacDonald R., Rutks I., Wilt T.J. (2015). Antimicrobial stewardship in outpatient settings: A systematic review. Infect. Control Hosp. Epidemiol..

[17] Meeker D., Knight T.K., Friedberg M.W., Linder J.A., Goldstein N.J., Fox C.R., Rothfeld A., Diaz G., Doctor J.N. (2014). Nudging guideline-concordant antibiotic prescribing: A randomized clinical trial. JAMA Intern. Med..

